# Persistence of Emotional Distress in Unaccompanied Migrant Children and Adolescents Primarily From the Northern Triangle of Central America

**DOI:** 10.1001/jamanetworkopen.2023.18977

**Published:** 2023-06-20

**Authors:** Natan J. Vega Potler, Jessica Zhang, Barbara Hackley, Jaeun Choi, Xianhong Xie, Brenda Punsky, Lisa Pineda, Alan Shapiro

**Affiliations:** 1 Department of Child and Adolescent Psychiatry, New York University Grossman School of Medicine, New York; 2 Department of Psychiatry, New York University Grossman School of Medicine, New York; 3 Bronx Health Collective, Montefiore Medical Center, Bronx, New York; 4 Department of Epidemiology and Population Health, Albert Einstein College of Medicine, Bronx, New York; 5 Department of Pediatrics, Montefiore Medical Center, Bronx, New York

## Abstract

**Importance:**

In the US, unaccompanied migrant children and adolescents (hereinafter referred to as children) are predominantly from Central America’s Northern Triangle. While unaccompanied migrant children are at high risk for psychiatric sequelae due to complex traumatic exposures, longitudinal investigations of psychiatric distress after resettlement are lacking.

**Objective:**

To identify factors associated with emotional distress and longitudinal changes in emotional distress among unaccompanied migrant children in the US.

**Design, Setting, and Participants:**

For this retrospective cohort study, the 15-item Refugee Health Screener (RHS-15) was administered between January 1, 2015, and December 31, 2019, to unaccompanied migrant children as part of their medical care to detect emotional distress. Follow-up RHS-15 results were included if they were completed before February 29, 2020. Median follow-up interval was 203 days (IQR, 113-375 days). The study was conducted in a federally qualified health center that provides medical, mental health, and legal services. Unaccompanied migrant children who completed the initial RHS-15 were eligible for analysis. Data were analyzed from April 18, 2022, to April 23, 2023.

**Exposures:**

Traumatic events before migration, during migration, during detention, and after resettlement in the US.

**Main Outcomes and Measures:**

Emotional distress, including symptoms of posttraumatic stress disorder, anxiety, and depressive symptoms, as indicated by the RHS-15 (ie, score ≥12 on items 1-14 or ≥5 on item 15).

**Results:**

In total, 176 unaccompanied migrant children completed an initial RHS-15. They were primarily from Central America’s Northern Triangle (153 [86.9%]), were mostly male (126 [71.6%]), and had a mean (SD) age of 16.9 (2.1) years. Of the 176 unaccompanied migrant children, 101 (57.4%) had screen results above the positive cutoff. Girls were more likely to have positive screen results than boys (odds ratio, 2.48 [95% CI, 1.15-5.34]; *P* = .02). Follow-up scores were available for 68 unaccompanied migrant children (38.6%). On the follow-up RHS-15, most scored above the positive cutoff (44 [64.7%]). Three-quarters of unaccompanied migrant children who scored above the positive cutoff initially continued to have positive scores at follow-up (30 of 40), and half of those with negative screen scores initially had positive scores at follow-up (14 of 28). Female vs male unaccompanied migrant children (unstandardized β = 5.14 [95% CI, 0.23-10.06]; *P* = .04) and initial total score (unstandardized β = 0.41 [95% CI, 0.18-0.64]; *P* = .001) were independently associated with increased follow-up RHS-15 total score.

**Conclusions and Relevance:**

The findings suggest that unaccompanied migrant children are at high risk for emotional distress, including symptoms of depression, anxiety, and posttraumatic stress. The persistence of emotional distress suggests that unaccompanied migrant children would benefit from ongoing psychosocial and material support after resettlement.

## Key Points

**Questions:**

How does emotional distress change longitudinally in unaccompanied migrant children and adolescents predominantly from the Northern Triangle of Central America, and what characteristics are associated with emotional distress?

**Findings:**

This cohort study of 176 unaccompanied migrant children and adolescents showed that most, particularly girls, report high rates of persistent emotional distress. Girls were more likely than boys to have increased severity of emotional distress at follow-up.

**Meaning:**

Results suggest that unaccompanied migrant children and adolescents, particularly girls, are at high risk for emotional distress that persists after resettlement in the US.

## Teaser

This cohort study identifies the rate of and factors associated with positive screening for emotional distress, changes in emotional distress, and characteristics associated with increased severity of emotional distress among unaccompanied migrant children primarily from the Northern Triangle of Central America.

## Introduction

*Unaccompanied migrant children* refers to children and adolescents younger than 18 years without legal status who migrate without a parent or guardian. Unaccompanied migrant children are often vulnerable to psychiatric sequelae because of traumatic events experienced at each stage of their migration journey.^1,2,3,4,5,6,7,8^ The migration of these children and adolescents is often precipitated by economic deprivation, climate disaster, and physical or sexual violence, including gang recruitment and violence^9,10,11^; during migration, these unaccompanied children and adolescents are vulnerable to robbery, extortion, kidnapping, and physical and sexual violence^4,11,12,13^; and after migration, many unaccompanied migrant children spend time in detention, which is associated with adverse mental and physical health consequences.^14,15,16,17,18^ In the US, unaccompanied migrant children are released from detention to the care of sponsors, including relatives or adults with whom they had no prior relationship.^10^ Afterward, many face linguistic and/or cultural barriers, discrimination, economic deprivation, community violence, and uncertainty about being granted immigration status.^4,19^

The global population of unaccompanied migrant children is increasing, and the United Nations Children’s Fund and High Commissioner for Refugees estimated that more than 300 000 unaccompanied migrant children migrated between 2015 and 2016 worldwide.^20^ In the US context, more than 171 000 unaccompanied migrant children were apprehended at the Mexican border in 2021,^21^ and more than 324 000 have been released to US-based sponsors during the last 4 fiscal years.^22^ Most unaccompanied children and adolescents who migrate to the US are male adolescents from the Northern Triangle countries of Central America: Guatemala, Honduras, and El Salvador. In fiscal year 2021, 92% of unaccompanied migrant children in the custody of the Office of Refugee Resettlement were from the Northern Triangle, 72% were older than 14 years, and 66% were boys.^23^

The 15-item Refugee Health Screener (RHS-15) was developed to address a lack of standardized questionnaires tailored to detect emotional distress relevant for culturally and linguistically diverse refugees and asylees.^24^ It probes posttraumatic stress disorder (PTSD), depression, and anxiety symptoms; shows good psychometric properties in multiethnic adults and adolescents, including unaccompanied migrant children; and has been translated into multiple languages.^25,26,27^ Higher scores and rates of distress on the RHS-15 are associated with older age,^28^ female sex,^28^ experiencing or witnessing violence,^28^ being denied asylum,^29^ and being an unaccompanied vs accompanied immigrant child.^29^ Despite an absence of guidelines on the frequency of repeated RHS-15 administration after resettlement, data indicate that screening at least twice may facilitate the identification of initial and delayed emotional distress.^30^ Studies of unaccompanied migrant children in Europe have characterized mental health symptoms and symptom chronicity,^31^ but these have limited applicability for unaccompanied migrant children in the US, given differences in country of origin, migration journey, and host country’s politicolegal context. No prior investigation, to our knowledge, has described the chronicity of emotional distress among unaccompanied migrant children resettled in the US. To address the gaps in the literature, this retrospective cohort study aimed to (1) identify the rate of and factors associated with positive emotional distress screening, as measured by the RHS-15, among unaccompanied migrant children primarily from the Northern Triangle; (2) describe longitudinal changes in emotional distress based on follow-up RHS-15; and (3) identify characteristics associated with increased severity.

## Methods

This study received approval and a waiver of informed consent from the institutional review board of Montefiore Medical Center. We followed the Strengthening the Reporting of Observational Studies in Epidemiology (STROBE) reporting guideline.

### Setting

Terra Firma was founded in 2013 as a holistic model of care integrating medical, mental health, and not-for-profit legal services to meet the complex needs of unaccompanied migrant children and is located in a Bronx-based federally qualified health center.^32^ Unaccompanied migrant children enter care at Terra Firma in 1 of 2 ways: (1) they are identified by a community-based organization and referred to Terra Firma or (2) they are identified by clinical staff at the health center and then internally referred to receive Terra Firma services. During the study period, a mean of 185 unique unaccompanied migrant children received services annually.

### Participants

To be eligible for this retrospective cohort study, patients had to be unaccompanied migrant children who completed an initial RHS-15 between January 1, 2015, and December 31, 2019, as part of their medical care at Terra Firma. Follow-up RHS-15 was included if completed before February 29, 2020 (ie, prior to the COVID-19 pandemic). During our study period, 443 unaccompanied migrant children were seen for at least 1 medical visit. Of the 226 unaccompanied migrant children who responded to the RHS-15, 176 (77.9%) completed it; 50 were excluded for having incomplete data.

### Variables and Measures

#### Participant Characteristics

Characteristics were selected based on previously identified risk factors for psychiatric symptoms among adolescent refugees, including age,^33^ biological sex,^34^ length of time after resettlement,^35^ preferred language, and country of origin.^27^ Data were recorded at entry to care at Terra Firma; they were extracted nonblinded by one of the authors (J.Z.) with graduate-level public health training. Data on race and ethnicity were obtained from the medical record, where it is collected at entry to care via self-report. Racism experienced at various stages of migration may contribute to emotional distress. Over 90% of the total sample either identified their race as “other” without specification or provided no race. Thus, methods of eliciting race and ethnicity among this clinical sample did not identify individuals of Indigenous heritage. Since many unaccompanied migrant children, particularly those from Guatemala, are Indigenous, this suggests that the exclusive use of US-centric racial and ethnic categories may contribute to the undercount of Indigenous migrants from Latin America.^36^ Date of birth and sex were extracted from the medical record using Clinical Looking Glass software (Montefiore Information Technology). Date of entry to the US, country of origin, and preferred language were extracted from the Terra Firma program registry, which is maintained by program administrators. Age at initial RHS-15 was calculated by subtracting date of birth from initial RHS-15 date and was used as a continuous variable. Country of origin, sex, and preferred language were used as categorical variables. Time in the US at initial RHS-15 was computed from the dates of entry to the US and RHS-15 administration and was categorized into tertiles (*≤*1 year, >1 to 2 years, and >2 years). The RHS-15 results and dates of administration were manually extracted from program records. Days between RHS-15 administrations were calculated from each date of service and used as a continuous variable. Characteristics of unaccompanied migrant children and RHS-15 data were merged by matching unique identifiers.

#### 15-Item Refugee Health Screener

The RHS-15 is completed at entry to care at Terra Firma and repeated at the discretion of medical and mental health care professionals. The RHS-15 was developed as a screening instrument of emotional distress, including symptoms of the common psychiatric conditions of major depression, anxiety, and PTSD, in refugee populations. Translation of the RHS-15 into available languages, including Spanish,^37^ has been performed in iterative, participatory processes, including refugees, experts, and professional translators to optimize the cultural responsiveness and linguistic relevance of the items. It has strong psychometric properties,^29,37,38^ including high sensitivity and specificity against validated diagnostic proxies of PTSD (0.81 and 0.87, respectively), anxiety (0.94 and 0.86, respectively), and depression (0.95 and 0.89, respectively).^24^ The 15-item version of the RHS-15 includes items from the Hopkins Symptoms Checklist–25, the New Mexico Refugee Symptom Checklist–121, and the Posttraumatic Symptoms Scale-Self Report, which were selected based on their association with PTSD, depression, and anxiety. Thirteen of the 15 items were taken from these instruments to measure the degree of distress associated with symptoms of PTSD, depression, and anxiety using a Likert response set ranging from not at all (0 points) to extremely bothersome (4 points); item 14 measures the degree to which individuals believe that they can cope using a Likert scale ranging from being able to cope with anything that comes your way (0 points) to unable to cope with anything (4 points); and item 15 is a distress thermometer ranging from 0 (no distress) to 10 (extreme distress). The RHS-15 total score is the sum of items 1 to 14 (score range, 0-56),^30^ and a positive RHS-15 result was defined as: (1) a total score of 12 or greater (of 56) or (2) a distress thermometer of 5 or greater (of 10), which is consistent with the cutoff found to optimize sensitivity and specificity for PTSD, depression, and anxiety.^24,37,39^

### Statistical Analysis

Data were analyzed from April 18, 2022, to April 23, 2023. Participant characteristics and initial and follow-up RHS-15 total scores were summarized as mean (SD) values for continuous variables and as frequencies and percentages for categorical variables. For comparison between unaccompanied migrant children with or without follow-up RHS-15, 2-sample *t* tests and χ^2^ tests were used for continuous and categorical variables, respectively. Only RHS-15 results with complete data on all 15 items were included in the analyses. To investigate differences in demographic characteristics between our study sample and excluded unaccompanied migrant children, χ^2^ and 2-sample *t* tests were used to compare between-group sex and age at entry to care.

Logistic regression was used to evaluate the unadjusted and adjusted associations of age, biological sex, and time in the US with positive initial RHS-15 results (ie, total score ≥12 and/or thermometer ≥5). For unaccompanied migrant children who completed a follow-up RHS-15, linear regression was used to investigate the unadjusted and adjusted associations of age, sex, time in the US, time between RHS-15 administrations, and initial RHS-15 total score with follow-up RHS-15 total score. Two-tailed *P* < .05 was considered statistically significant. Data were analyzed using SAS software, version 9.4 (SAS Institute, Inc).

## Results

### Sample Characteristics

Characteristics of the total sample of 176 unaccompanied migrant children included in our initial analysis are displayed in Table 1. Unaccompanied migrant children were primarily male adolescents (126 [71.6%] vs 50 [28.4%] female; mean [SD] age, 16.9 [2.1] years) from Central America’s Northern Triangle countries of Honduras, Guatemala, and El Salvador (153 [86.9%]). The remaining 23 unaccompanied migrant children were born elsewhere in Latin America and the Caribbean (Mexico, Ecuador, Colombia, Nicaragua, or Dominican Republic) or in African countries (Ghana, Sierra Leone, The Gambia, or Somalia). In terms of race, 10 unaccompanied migrant children (5.7%) self identified as Black or African American, 4 (2.3%) as White, and 143 (81.3%) as other without further specification; 19 (10.8%) either declined to respond or did not respond. With respect to ethnicity, 136 (73.3%) were Hispanic/Latino, 5 (2.8%) were Non-Hispanic/Latino, 10 (5.7%) specified a Latin American country (eg, Honduran), and 25 (14.2%) either declined to or did not respond. Of the 176 unaccompanied migrant children, 105 (59.7%) had been in the US for 2 years or less at the time of first emotional distress screening. Table 1 also displays unaccompanied migrant children included in the follow-up analysis.

**Table 1.** Characteristics of All Unaccompanied Migrant Children and Adolescents Who Completed Initial and Follow-up RHS-15^a^

Characteristic	Total sample (n = 176)	Sample with a follow-up RHS-15 (n = 68)
Age at initial RHS-15, mean (SD), y	16.9 (2.1)	16.4 (1.7)
Sex		
Male	126 (71.6)	42 (61.8)
Female	50 (28.4)	26 (38.2)
Country or region of origin		
Honduras	97 (55.1)	41 (60.3)
Guatemala	34 (19.3)	16 (23.5)
El Salvador	22 (12.5)	8 (11.8)
Other Latin American or Caribbean^b^	17 (9.7)	1 (1.5)
Africa^c^	6 (3.4)	2 (2.9)
Race		
Black or African American	10 (5.7)	4 (5.9)
White	4 (2.3)	0
Other^d^	143 (81.3)	60 (88.2)
Declined or did not respond	19 (10.8)	4 (5.9)
Ethnicity		
Hispanic/Latino	136 (73.3)	60 (88.2)
Non-Hispanic/Latino	5 (2.8)	2 (2.9)
Specified Latin American country	10 (5.7)	2 (2.9)
Declined or did not respond	25 (14.2)	4 (5.9)
Preferred language		
Spanish	168 (95.5)	66 (97.1)
English	4 (2.3)	1 (1.5)
Other	4 (2.3)	1 (1.5)
Time in US at initial RHS-15, y^e^		
≤1	63 (43.4)	35 (57.4)
>1 to 2	42 (29.0)	16 (26.2)
>2	40 (27.6)	10 (16.4)

Abbreviation: RHS-15, 15-item Refugee Health Screener.

^a^ Unless otherwise indicated, data are expressed as No. (%) of participants. Percentages have been rounded and may not total 100.

^b^ Includes Mexico, Ecuador, Colombia, Nicaragua, Dominican Republic, and Jamaica.

^c^ Includes Ghana, Sierra Leone, The Gambia, and Somalia.

^d^ Race not further specified.

^e^ Thirty-one participants were missing data in the total sample and 7 in the follow-up sample.

### Initial Assessment of Rate of Emotional Distress and Associated Characteristics

On initial RHS-15 assessment, most unaccompanied migrant children (101 [57.4%]) screened above the positive cutoff for emotional distress (ie, total score ≥12 and/or thermometer ≥5). Eighty-seven unaccompanied migrant children (49.4%) had a total score of 12 or greater, 72 (40.9%) had a distress thermometer score of 5 or greater, and 58 (33.0%) had positive screen results on both criteria. The rate of initial RHS-15 positivity was higher for girls (35 [70.0%]) than boys (66 [52.4%]). The mean (SD) RHS-15 total score was 13.9 (11.0), and the median was 11 (IQR, 5-21). As shown in Table 2, multivariable logistic regression demonstrated an independent association of sex with emotional distress positivity, with girls having more than double the odds of a positive RHS-15 screen result for emotional distress (odds ratio, 2.48 [95% CI, 1.15-5.34]; *P* = .02). Neither age nor time in the US was associated with a positive emotional distress screen result on the initial RHS-15.

**Table 2.** Logistic Regression Analysis on Initial RHS-15 Score Above the Positive Cutoff

Variable	Positive result on initial RHS-15, No. (%)	Univariable analysis		Multivariable analysis	
		Odds ratio (95% CI)	*P* value	Odds ratio (95% CI)	*P* value
Age, y	NA	1.14 (0.98-1.32)	.09	1.14 (0.95-1.37)	.17
Sex					
Male	66 (52.4)	1 [Reference]	NA	1 [Reference]	NA
Female	35 (70.0)	2.12 (1.06-4.27)	.03	2.48 (1.15-5.34)	.02
Time in US at initial RHS-15, y					
≤1	31 (49.2)	1 [Reference]	NA	1 [Reference]	NA
>1 to 2	22 (52.4)	1.14 (0.52-2.48)	.75	1.13 (0.51-2.52)	.77
>2	26 (65.0)	1.92 (0.85-4.34)	.12	1.50 (0.63-3.56)	.36

Abbreviations: NA, not applicable; RHS-15, 15-item Refugee Health Screener.

### Follow-up Assessment

A follow-up assessment was available for 68 of 176 unaccompanied migrant children (38.6%). The median time between initial and follow-up RHS-15 was almost 7 months (203 days [IQR, 113-375 days]). Eighteen unaccompanied migrant children (26.5%) completed a follow-up RHS-15 after an interval of 1 year or longer.

#### Characteristics of Unaccompanied Migrant Children With Follow-up Assessment

The rates of positivity on initial RHS-15 between unaccompanied migrant children did not differ significantly between those who completed a follow-up (n = 68) and those who did not (n = 108) (40 [58.8%] vs 61 [56.5%], respectively; *P* = .76). Unaccompanied migrant children who completed a follow-up RHS-15 were significantly younger (mean [SD] age, 16.4 [1.7] vs 17.2 [2.2] years; *P* = .01) and more likely to be girls (26 [38.2%] vs 24 [22.2%]; *P* = .02) compared with those who only completed an initial RHS-15. Unaccompanied migrant children who completed a follow-up RHS-15 had been in the US for less time than those who only completed an initial RHS-15 (51 [75.0%] had arrived within 2 years vs 54 [50.0%]; *P* = .01) (eTable in Supplement 1).

#### Longitudinal Changes in Emotional Distress

On follow-up assessment, 44 unaccompanied migrant children (64.7%) scored above the cutoff. The mean (SD) total score for unaccompanied migrant children was 16.4 (10.5), and the median was 16 (IQR, 7-24). The mean (SD) change from initial to follow-up RHS-15 was an increase of 2.0 (10.0). The Figure displays longitudinal changes in emotional distress screening. Three-quarters of those who initially scored above the positive cutoff remained positive at follow-up (30 of 40). In contrast, half of those who scored below the cutoff on initial RHS-15 scored above the cutoff at follow-up (14 of 28). Compared with unaccompanied migrant children who scored above the cutoff at initial and follow-up RHS-15, those who scored below the cutoff on both were younger (mean [SD] age, 15.6 [1.4] vs 17.1 [1.6] years; *P* = .03).

**Figure.**
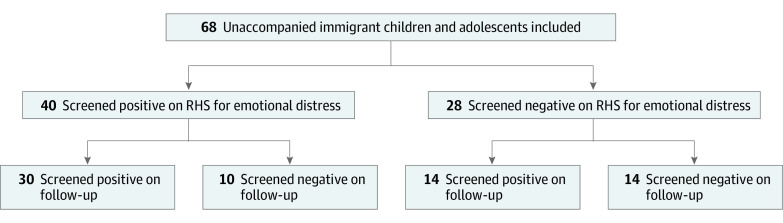
Emotional Distress Positivity on Initial and Follow-up 15-Item Refugee Health Screener (RHS-15) RHS-15 scores above the positive cutoff (total score ≥12 and/or thermometer ≥5) at initial and follow-up administration among unaccompanied migrant children and adolescents are shown.

#### Characteristics Associated With Emotional Distress Severity at Follow-up

Multivariable linear regression was conducted to investigate the associations of the characteristics of unaccompanied migrant children and the initial RHS-15 total score with the follow-up RHS-15 total score (Table 3). Independent associations were observed for girls vs boys (unstandardized β = 5.14 [95% CI, 0.23-10.06]; *P* = .04) and initial RHS-15 total score (unstandardized β = 0.41 [95% CI, 0.18-0.64]; *P* = .001) with higher follow-up RHS-15 total score. No associations were observed between follow-up RHS-15 total score and age, time in the US at initial RHS-15, or follow-up interval.

**Table 3.** Linear Regression Analysis on Follow-up Total RHS-15 Score

Variable	Mean (SD) follow-up RHS-15 score^a^	Univariable analysis		Multivariable analysis	
		Unstandardized β (95% CI)	*P* value	Unstandardized β (95% CI)	*P* value
Initial RHS-15 total score	NA	0.54 (0.35 to 0.73)	<.001	0.41 (0.18 to 0.64)	.001
Age at first screen, y	NA	1.15 (−0.28 to 2.58)	.11	1.17 (−0.31 to 2.65)	.12
Sex					
Male	14.19 (10.27)	1 [Reference]	NA	1 [Reference]	NA
Female	19.96 (10.13)	5.77 (0.85 to 10.69)	.02	5.14 (0.23 to 10.06)	.04
Time in US at first screen, y					
≤1	15.66 (10.88)	1 [Reference]	NA	1 [Reference]	NA
>1 to 2	15.13 (9.68)	−0.53 (−6.57 to 5.51)	.86	−0.13 (−5.53 to 5.26)	.96
>2	22.90 (10.17)	7.24 (0.07 to 14.42)	.05	0.69 (−5.77 to 7.14)	.83
Time from initial RHS-15 to follow-up, d	NA	−0.001 (−0.011 to 0.009)	.81	0.004 (−0.005 to 0.012)	.43

Abbreviations: NA, not applicable; RHS-15, 15-item Refugee Health Screener.

^a^ Positive cutoff score is a total score of 12 or greater (of 56) or (2) a distress thermometer of 5 or greater (of 10).

#### Characteristics of Unaccompanied Migrant Children With Missing and Incomplete RHS-15 Data

The proportion of female unaccompanied migrant children analyzed was lower than among those who were excluded (50 [28.4%] vs 81 [37.3%]), although this did not reach statistical significance (χ^2^_1_ = 3.48 [n = 443]; *P* = .06). Similarly, age at entry to care did not differ significantly between analyzed and excluded unaccompanied migrant children (mean [SD] age, 16.6 [2.1] vs 16.6 [2.9] years; *P* = .89).

## Discussion

The present investigation examined emotional distress, its associated characteristics, and longitudinal changes in unaccompanied migrant children after resettlement in the US while they received care in a wrap-around service program. The demographic characteristics of the study cohort closely parallel the population of unaccompanied migrant children in the US (ie, primarily male adolescents from Central America’s Northern Triangle). As a result, the findings contribute to a much-needed literature on the rising population of unaccompanied migrant children who migrate to the US.

Most of the cohort screened above the cutoff for emotional distress, suggesting that they may experience PTSD, depression, and/or anxiety symptoms. RHS-15 positivity rates for unaccompanied migrant children in this sample were comparable with those of many adult refugees^40^ and unaccompanied refugee youths resettled in Europe.^27^ In addition to high rates of emotional distress among unaccompanied migrant children overall, the odds of reporting emotional distress were twice as high for girls compared with boys on the initial RHS-15. This is similar to findings from unaccompanied migrant children from non–Northern Triangle countries that girls report elevated psychiatric symptoms compared with boys.^1,19,20,41^ Investigations have also found an association between older age and increased psychiatric symptoms in unaccompanied migrant children.^27,33^ We did not detect such an association in this sample, perhaps because of relatively low statistical power.

Longitudinally, distress related to PTSD, depression, and/or anxiety appeared to persist or worsen from initial to follow-up RHS-15. Such findings are consistent with longitudinal observations of multiethnic unaccompanied migrant children who resettled in Europe, despite notable differences in country of origin, migration journey, and the host country’s politicolegal context.^19,31,35^ In addition to showing that mental health symptoms endure, these studies have elucidated several protective and risk factors. For example, while being granted asylum is associated with improving mental health outcomes,^29,42^ uncertainty about or denial of legal status,^31,42^ increased traumatic events,^19,34^ psychosocial stressors (eg, economic stress, discrimination, acculturative stress),^19,34^ older age,^34,35^ and female sex^34^ are associated with more persistent symptom severity. While comparable studies for unaccompanied migrant children from the Northern Triangle are lacking, such factors may have contributed to the present findings of persistent emotional distress. The sampling interval between initial and follow-up RHS-15 may have also contributed to our results. While some trauma-related symptoms may remit within a year,^43^ they may persist longer for migrant and refugee youths, especially when, as with unaccompanied migrant children, they confront ongoing stressors.^35,44^ Although most unaccompanied migrant children reported persistent psychiatric symptoms, more than one-fifth scored below the cutoff at initial and follow-up administration. This may reflect the strength and resilience of unaccompanied migrant children, as highlighted elsewhere.^45,46,47^

Another notable result was that female sex was associated with higher odds of scoring above the cutoff on the initial RHS-15, as well as with increased follow-up RHS-15 score. Female vs male sex was associated with a more than 5-point increase in total follow-up RHS-15, notable given the positive cutoff value of 12 or higher. These results are comparable to studies of adolescent refugees and asylees from other countries of origin, which have found that girls report higher levels of psychiatric symptoms compared with boys.^20,34,41^ A possible contributor for elevated risk of emotional distress for female unaccompanied children and adolescents migrating to the US is the high rates of interpersonal trauma, including sexual violence experienced by girls and women before and during migration.^11,20^ Alternatively, this may be related to findings from multiethnic adolescents that coping strategies can vary by gender and sex^48,49,50,51,52^ and that some strategies (eg, avoidance)^53^ may limit reported psychiatric symptoms. Social expectations may also influence disclosure of distress (eg, boys may underreport symptoms).^54^ This cohort study is notable because it describes longitudinal emotional distress in unaccompanied migrant children predominantly from the Northern Triangle—where most unaccompanied migrant children in the US originate—and examines demographic characteristics associated with psychiatric symptoms in one of the largest community samples resettled in the US. In noting the mental health emergency at the Southwest border, clinicians and advocates have emphasized the importance of mitigating further traumatization of unaccompanied migrant children by immigration policy, including the abolition of immigrant detention^55,56,57,58,59^ and increasing funding for resettlement services.^10^

### Limitations

Study limitations include the generalizability of findings to unaccompanied migrant children who do not remain connected to clinical care, since this sample consisted of youth receiving care in a wrap-around service model. Thus, the findings are likely conservative; other unaccompanied migrant children may experience even higher levels of emotional distress. Although our analyses did not include all unaccompanied migrant children at Terra Firma, we observed no statistically significant differences in sex and age at entry to care among our sample compared with excluded unaccompanied migrant children. The lower proportion of girls in our sample, however, suggests that our results may have underestimated emotional distress prevalence. Analyses were unable to consider other possible protective or risk factors, including coping strategies, psychosocial support, specific mental health services received, sponsor identity, immigration status changes, and level of economic deprivation in the resettled community, because such data were not collected. Additionally, the entire cohort of unaccompanied migrant children with an initial RHS-15 assessment did not complete a follow-up RHS-15 within the study period, which was truncated by the pandemic; the higher proportion of female unaccompanied migrant children among those with follow-up may have contributed to higher positive RHS-15 rates. Furthermore, as the RHS-15 is only validated as a screener, diagnostic prevalence rates of depression, anxiety, or PTSD could not be obtained.

## Conclusions

The present findings indicate that unaccompanied migrant children, particularly girls, experience persistent psychiatric symptoms after resettlement, suggesting that they would benefit from ongoing interdisciplinary support and monitoring for emotional distress. More extensive longitudinal studies that identify areas of resilience, protective factors, and additional risk factors for persistent affective distress are critical to support the mental health of unaccompanied migrant children after resettlement at the clinical, program, and policy levels.

## Notes

Supplement 1. **eTable.** Comparison of Unaccompanied Migrant Children Who Completed Only Initial vs Initial and Follow-up RHS-15

Supplement 2.
Data Sharing Statement
