# Supplementary material for: Persistence of Emotional Distress in Unaccompanied Migrant Children and Adolescents Primarily From the Northern Triangle of Central America
Source: JAMA Netw Open. 2023 Jun 20;6(6):e2318977. doi: 10.1001/jamanetworkopen.2023.18977 (PMC10282890; doi:10.1001/jamanetworkopen.2023.18977)
Supplement: Supplement 1. — eTable. Comparison of Unaccompanied Migrant Children Who Completed Only Initial vs Initial and Follow-up RHS-15 [file jamanetwopen-e2318977-s001.pdf]

# Supplementary Online Content

Vega Potler NJ, Zhang J, Hackley B, et al. Persistence of emotional distress in unaccompanied migrant children and adolescents primarily from the Northern Triangle of Central America. *JAMA Netw Open*. 2023;6(6):e2318977. doi:10.1001/jamanetworkopen.2023.18977

**eTable.** Comparison of Unaccompanied Migrant Children Who Completed Only Initial vs Initial and Follow-up RHS-15

This supplementary material has been provided by the authors to give readers additional information about their work.

©2023 Vega Potler NJ et al. *JAMA Network Open*.

**eTable.** Comparison of Unaccompanied Migrant Children Who Completed Only Initial vs Initial and Follow-up RHS-15

| **Variable** | **Number of RHS-15** | | **P-value** |
|---|---|---|---|
| | **Completed only Initial RHS-15 (n = 108)** | **Completed Follow-up RHS-15 (n = 68)** | |
| Age at initial RHS-15 (Years), mean (SD) | 17.2 (2.2) | 16.4 (1.7) | **.01** |
| Sex, n (%) | | | **.02** |
| Male | 84 (77.8) | 42 (61.8) | |
| Female | 24 (22.2) | 26 (38.2) | |
| Time in US at initial RHS-15, n (%) | | | **.01** |
| 1 year or less | 28 (33.3) | 35 (57.4) | |
| >1 to 2 years | 26 (31.0) | 16 (26.2) | |
| Over 2 years | 30 (35.7) | 10 (16.4) | |
| Initial RHS-15 above the cutoff, n (%) | | | .76 |
| Negative (below) | 47 (43.5) | 28 (41.2) | |
| Positive (above) | 61 (56.5) | 40 (58.8) | |

Abbreviation: RHS-15, 15-item Refugee Health Screener

©2023 Vega Potler NJ et al. *JAMA Network Open*.
