# Supplementary material for: Persistence of Emotional Distress in Unaccompanied Migrant Children and Adolescents Primarily From the Northern Triangle of Central America
Source: JAMA Netw Open. 2023 Jun 20;6(6):e2318977. doi: 10.1001/jamanetworkopen.2023.18977 (PMC10282890; doi:10.1001/jamanetworkopen.2023.18977)
Supplement: Supplement 2. — Data Sharing Statement [file jamanetwopen-e2318977-s002.pdf]

Data Sharing Statement

Vega Potler. Persistence of Emotional Distress in Unaccompanied Migrant Children and Adolescents Primarily From the Northern Triangle of Central America. *JAMA Netw Open*. Published June 20, 2023. doi:10.1001/jamanetworkopen.2023.18977

Data
Data available: No
